# Supplementary material for: When does post-ingestive dopamine signaling occur in humans?
Source: bioRxiv. 2026 Oct 1:2026.09.25.754497. Preprint. [Version 1] doi: 10.64898/2026.09.25.754497 (PMC13648475; doi:10.64898/2026.09.25.754497)
Supplement: Supplementary file 1 [file NIHPP2026.09.25.754497v1-supplement-1.pdf]

bioRxiv preprint doi: https://doi.org/10.64898/2026.09.25.754497; this version posted October 1, 2026. The copyright holder for this preprint (which was not certified by peer review) is the author/funder. This article is a US Government work. It is not subject to copyright under 17 USC 105 and is also made available for use under a CC0 license.

## SUPPLEMENTARY INFORMATION

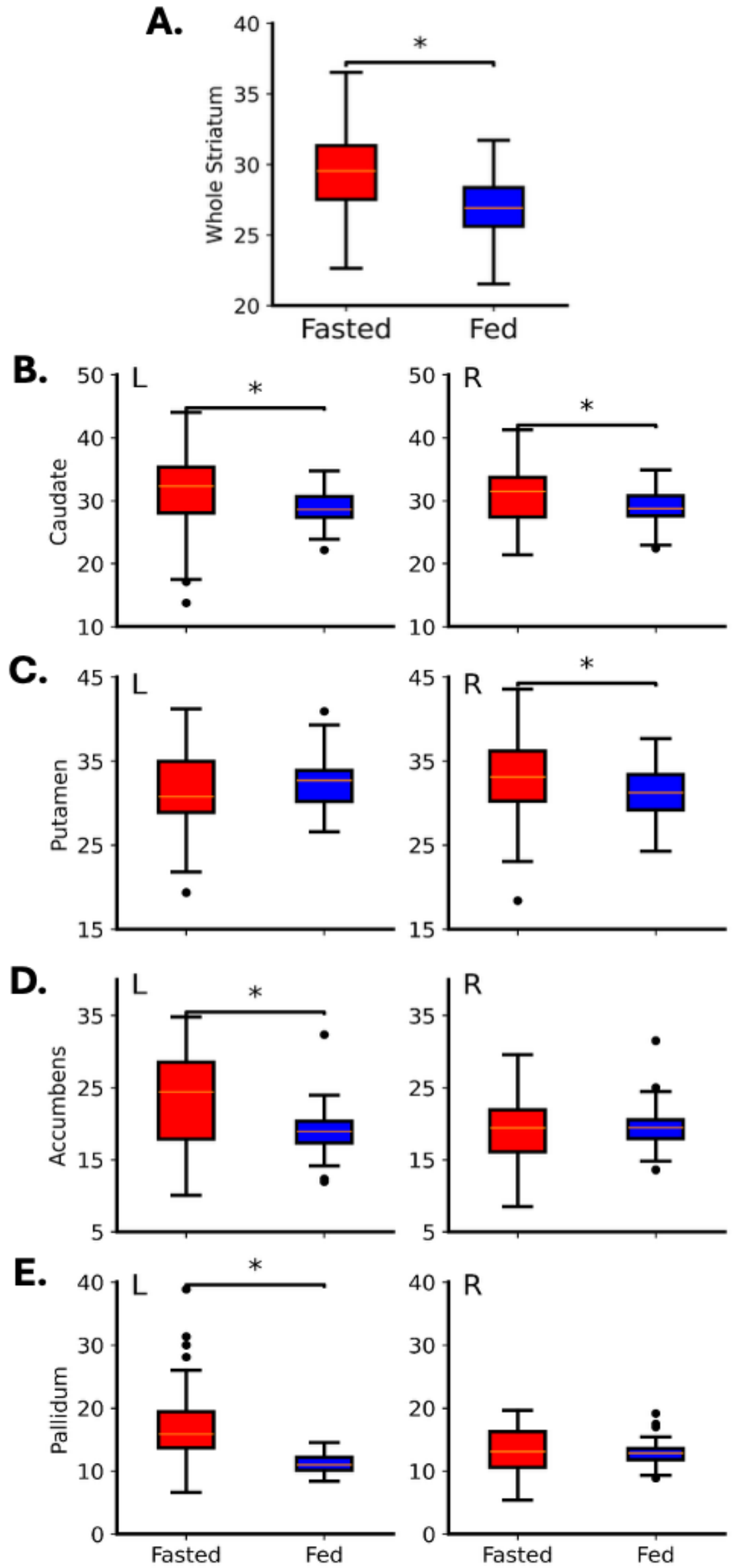


**Supplementary Figure 1. Comparisons of striatal dopamine D2R binding potential in ROIs between Fed vs Fasted Cohorts adjusted for age, sex, and HOMA-IR.** Dopamine D2R binding potential measured by [18F]fallypride is significantly reduced in individuals in the 2-hour post-ingestive state (Fed, n=43; blue bars) compared to individuals in the overnight fasted state (Fasted n=57; red bars) The differences in binding potential (mean±SEM) were (A) whole striatum, -2.93 ± 0.68 ($p < 0.0001$), (B) caudate (left, -2.5 ± 1.06 ($p = 0.02$); right, -2.01 ± 0.9 ($p = 0.03$)), (C) putamen (left, 1 ± 0.93 ($p = 0.29$); right, -1.93 ± 0.96 ($p = 0.047$)), (D) accumbens (left, -4.36 ± 1.21 ($p = 0.0006$); right, 0.77 ± 0.96 ($p = 0.42$)) and (E) pallidum (left, -5.86 ± 1.03 ($p < 0.001$); right, -0.21 ± 0.66 ($p = 0.75$)).

bioRxiv preprint doi: https://doi.org/10.64898/2026.09.25.754497; this version posted October 1, 2026. The copyright holder for this preprint (which was not certified by peer review) is the author/funder. This article is a US Government work. It is not subject to copyright under 17 USC 105 and is also made available for use under a CC0 license.

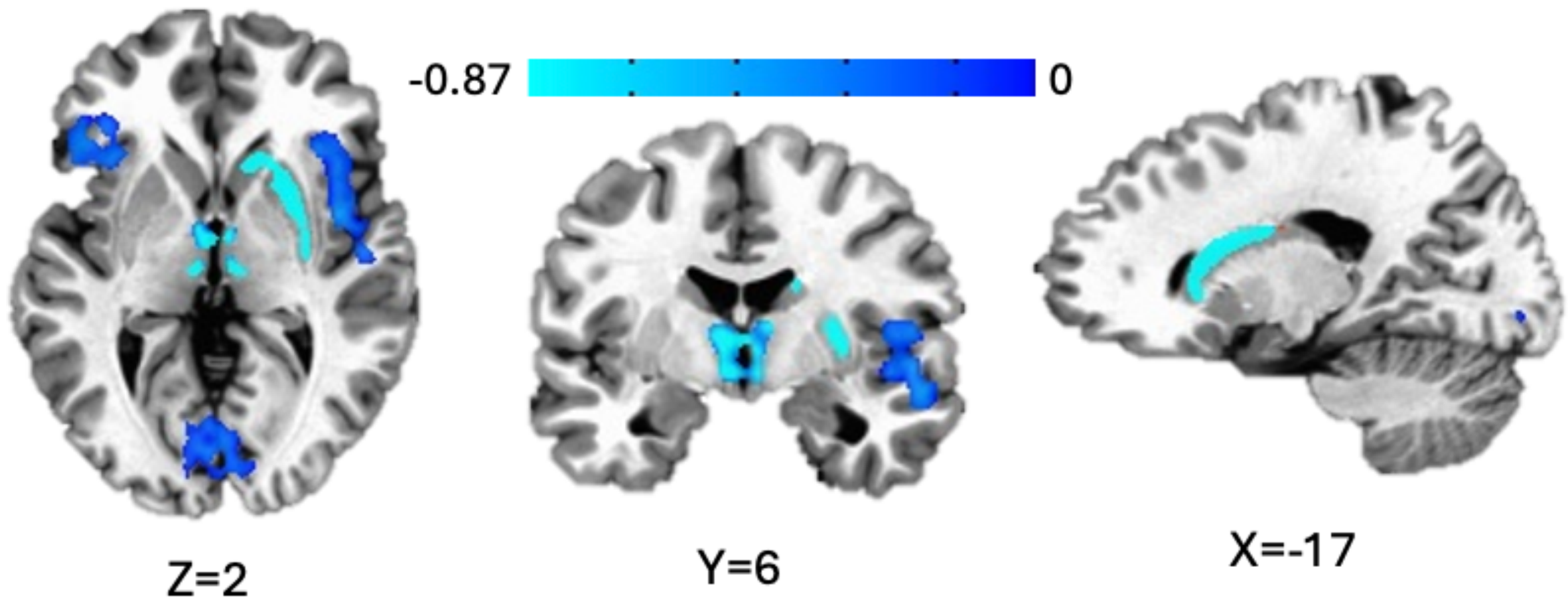


**Supplementary figure 2.** Voxel-wise comparisons of striatal dopamine D2R binding in fed vs. fasting state cohorts after adjusting for age, sex, and insulin sensitivity (HOMA-IR). Voxel-wise analyses identified clusters across the whole brain where dopamine D2R binding potentials significantly decreased in fed state compared to fasting state Clusters are defined by faces-touching neighbor clustering, bi-sided uncorrected $p < 0.0001$, cluster size > 30 voxels, and multiple-comparison corrected $p < 0.05$. Voxels are displayed after cubic convolution resampling. The color map represents the reduced BP's in individual voxels.

bioRxiv preprint doi: https://doi.org/10.64898/2026.09.25.754497; this version posted October 1, 2026. The copyright holder for this preprint (which was not certified by peer review) is the author/funder. This article is a US Government work. It is not subject to copyright under 17 USC 105 and is also made available for use under a CC0 license.

**Supplementary Table 1.** Locations of clusters from voxelwise analyses after adjusting for age sex and insulin sensitivity (HOMA-IR). PET resolution 3.5mm$^3$. Imaging analyses conducted in Analysis of Functional Neuroimaging (AFNI) (clusters defined by voxels with faces touching). Coordinates in Talairach Space.

| | Location of Peak | | | Voxels | Size | t-stat | alpha |
|---|---|---|---|---|---|---|---|
| | x | y | z | | (mm3) | | |
| **Comparisons of BPs adjusted for age sex and HOMA** | | | | | | | |
| Right insula | -54.2 | 18.5 | -4 | 170 | 7289 | -4.45 | <0.001 |
| Right Inferior frontal gyrus (pars Orbitalis) | | | | | | | |
| Right superior temporal gyrus | | | | | | | |
| Left calcarine gyrus | 1.8 | 78 | 3 | 117 | 5016 | -6.71 | <0.001 |
| Right calcarine gyrus | | | | | | | |
| Left lingual gyrus | | | | | | | |
| Left inferior frontal gyrus (pars Triangularis) | 47.2 | -23.5 | 3 | 84 | 3602 | -6.74 | <0.001 |
| Left inferior frontal gyrus (pars Orbitalis) | | | | | | | |
| Left insula | | | | | | | |
| Left thalamus | 1.8 | 8 | 6.5 | 51 | 2187 | -7.85 | <0.001 |
| Right thalamus | | | | | | | |
| Left ventral diencephalon | | | | | | | |
| Right putamen | -29.8 | 8 | 3 | 36 | 1544 | -5.99 | <0.001 |
| Right pallidum | | | | | | | |
| Right caudate | -15.8 | -13.0 | 17 | 32 | 1372 | -6.37 | <0.001 |
| Right putamen | | | | | | | |
| **Comparisons of BMI slopes adjusted for age sex and HOMA** | | | | | | | |
| Right caudate | -15.8 | -20 | 6.5 | 57 | 2444 | 2.70 | <0.005 |
| Right putamen | | | | | | | |
| Right olfactory cortex | | | | | | | |
| Left putamen | 19.2 | -16.5 | -0.5 | 48 | 2058 | 2.58 | <0.005 |
| Left insula | | | | | | | |
| Left olfactory cortex | | | | | | | |

*Continued below.*

bioRxiv preprint doi: https://doi.org/10.64898/2026.09.25.754497; this version posted October 1, 2026. The copyright holder for this preprint (which was not certified by peer review) is the author/funder. This article is a US Government work. It is not subject to copyright under 17 USC 105 and is also made available for use under a CC0 license.

| | Location of Peak | | | Voxels | Size | t-stat | alpha |
|---|---|---|---|---|---|---|---|
| | x | y | z | | (mm3) | | |
| **Difference Between Cohorts, adjusted for age, sex and HOMA-IR (D2BP Fed – D2BP Fasted)[#]** | | | | | | | |
| Insula, right | -54.2 | 18.5 | -4 | 170 | 7289 | -4.45 | <0.001 |
| Inferior frontal gyrus (pars Orbitalis), right | | | | | | | |
| Superior temporal gyrus, right | | | | | | | |
| Calcarine gyrus, left | 1.8 | 78 | 3 | 117 | 5016 | -6.71 | <0.001 |
| Calcarine gyrus, right | | | | | | | |
| Lingual gyrus, left | | | | | | | |
| Inferior frontal gyrus (pars Triangularis), left | 47.2 | -23.5 | 3 | 84 | 3602 | -6.74 | <0.001 |
| Inferior frontal gyrus (pars Orbitalis), left | | | | | | | |
| Insula, left | | | | | | | |
| Thalamus, left | 1.8 | 8 | 6.5 | 51 | 2187 | -7.85 | <0.001 |
| Thalamus, right | | | | | | | |
| Ventral diencephalon, left | | | | | | | |
| Putamen, right | -29.8 | 8 | 3 | 36 | 1544 | -5.99 | <0.001 |
| Pallidum, right | | | | | | | |
| Caudate, right | -15.8 | -13.0 | 17 | 32 | 1372 | -6.37 | <0.001 |
| Putamen, right | | | | | | | |
| **Comparisons of BMI slopes adjusted for age sex and HOMA-IR (β Fed – β Fasted)[*]** | | | | | | | |
| Caudate, right | -15.8 | -20 | 6.5 | 57 | 2444 | 2.70 | <0.005 |
| Putamen, right | | | | | | | |
| Olfactory cortex, right | | | | | | | |
| Putamen, left | 19.2 | -16.5 | -0.5 | 48 | 2058 | 2.58 | <0.005 |
| Insula, left | | | | | | | |
| Olfactory cortex, left | | | | | | | |

*Maximum of top three descriptive labels reflecting largest proportion of cluster provided using the atlas accounting for greatest percent of anatomical localization of cluster.*
*# Clusters are defined by faces-touching neighbor clustering, bi-sided uncorrected $p < 0.0001$, cluster size > 30 voxels, and multiple-comparison corrected $p < 0.05$.*
** Clusters are defined by faces-touching neighbor clustering, bi-sided uncorrected $p < 0.1$, cluster size > 30 voxels, and multiple-comparison corrected $p < 0.05$.*

bioRxiv preprint doi: https://doi.org/10.64898/2026.09.25.754497; this version posted October 1, 2026. The copyright holder for this preprint (which was not certified by peer review) is the author/funder. This article is a US Government work. It is not subject to copyright under 17 USC 105 and is also made available for use under a CC0 license.

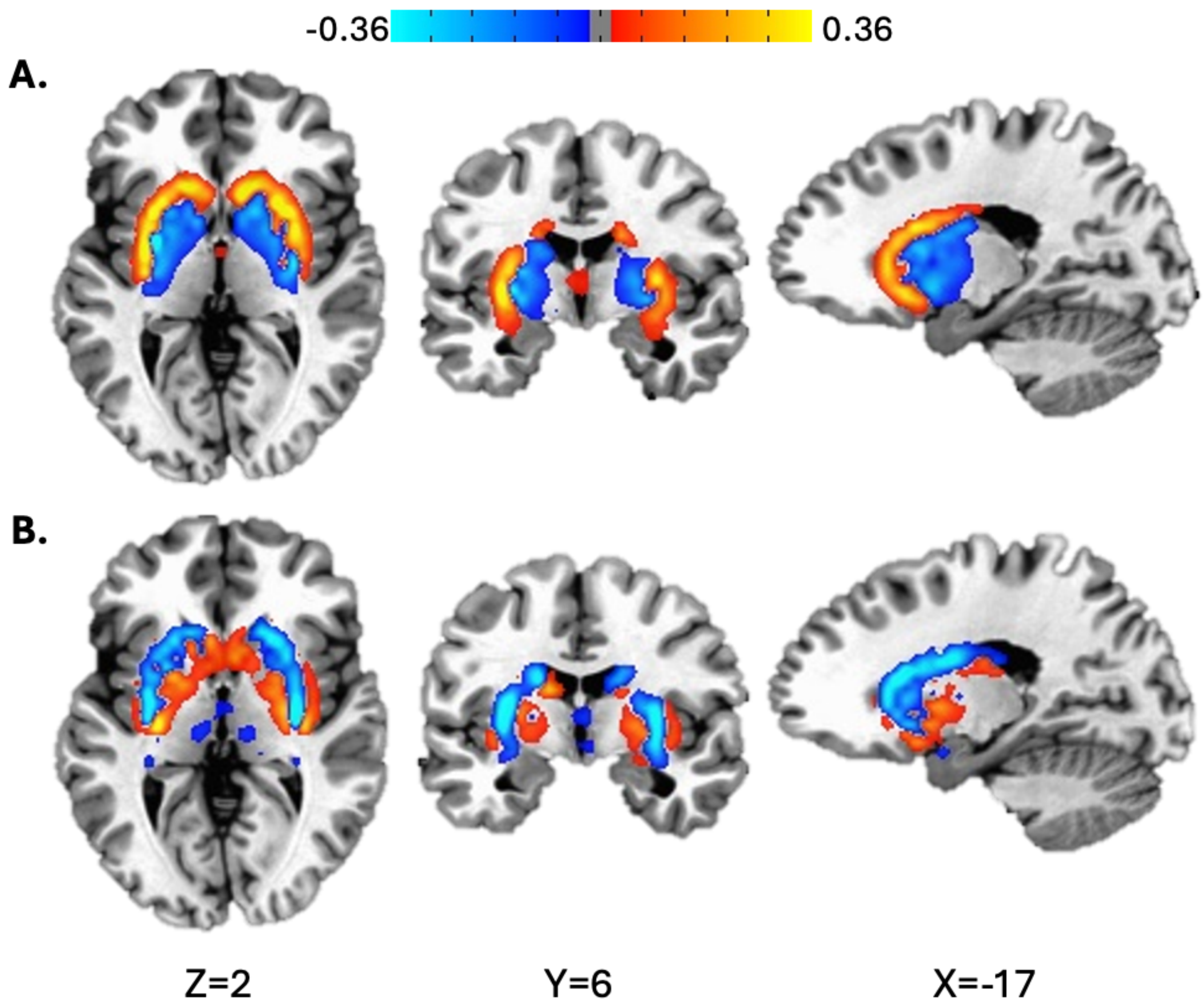


**Supplementary figure 3.** Unthresholded voxel-wise correlation patterns between dopamine D2R binding and BMI in **(A)** Fed Cohort and **(B)** Fasted Cohort. Regions where D2BP increases with BMI (suggestive of reduced synaptic dopamine) indicated in red/orange, while regions where D2BP decreases with BMI (suggestive of increased synaptic dopamine) indicated in blues. Clusters are defined by faces-touching neighbor clustering, bi-sided uncorrected $p < 0.0001$, cluster size > 30 voxels. Clusters uncorrected for multiple comparisons for visualization purposes. Voxels are displayed after cubic convolution resampling.

bioRxiv preprint doi: https://doi.org/10.64898/2026.09.25.754497; this version posted October 1, 2026. The copyright holder for this preprint (which was not certified by peer review) is the author/funder. This article is a US Government work. It is not subject to copyright under 17 USC 105 and is also made available for use under a CC0 license.

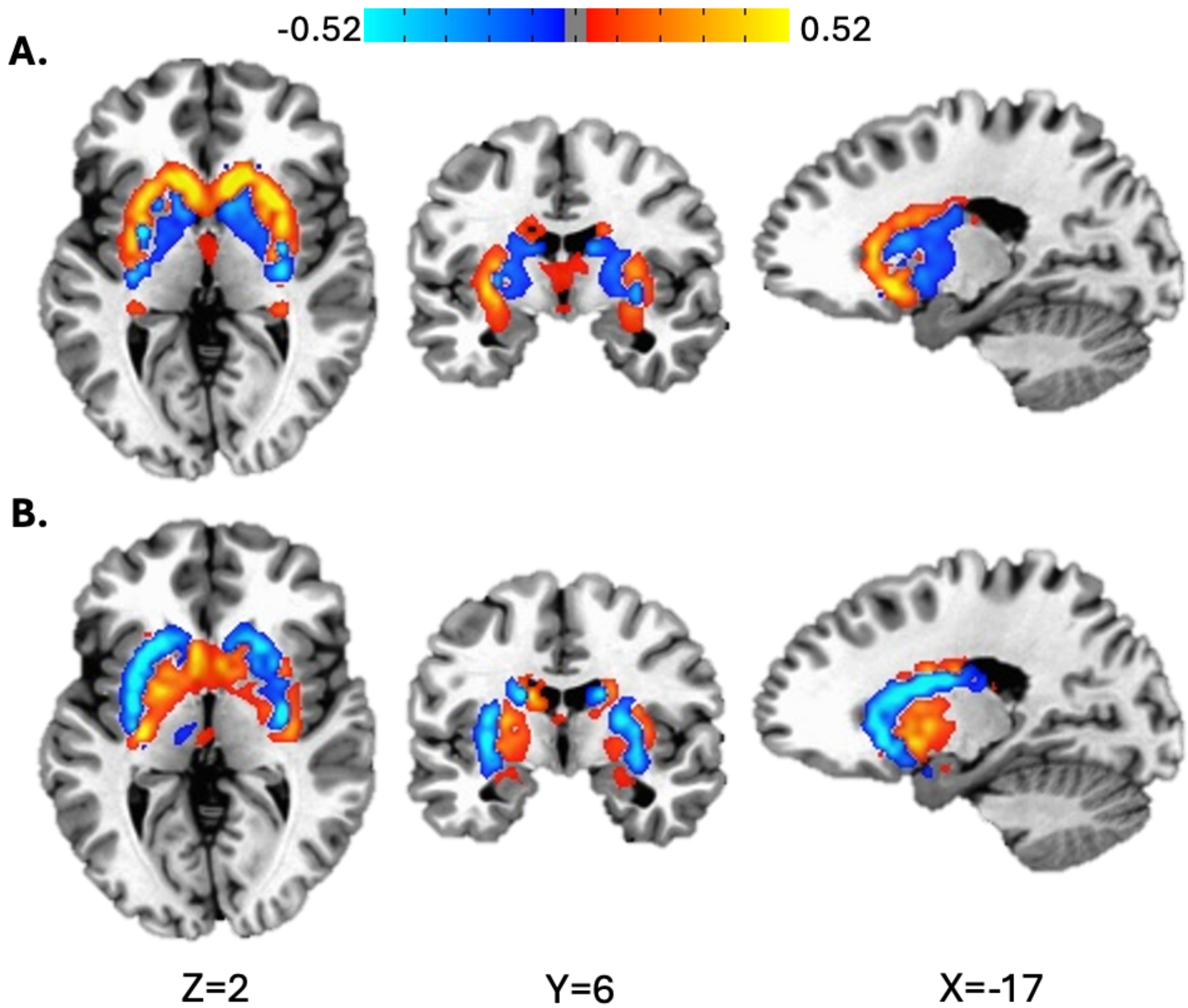


**Supplementary figure 4.** Unthresholded voxel-wise correlation patterns between dopamine D2R binding and BMI *adjusted for age, sex, and HOMA-IR* in **(A)** Fed Cohort and **(B)** Fasted Cohort. Regions where D2BP increases with BMI (suggestive of reduced synaptic dopamine) indicated in red/orange, while regions where D2BP decreases with BMI (suggestive of increased synaptic dopamine) indicated in blues. Clusters are defined by faces-touching neighbor clustering, bi-sided uncorrected $p < 0.0001$, cluster size > 30 voxels. Clusters uncorrected for multiple comparisons for visualization purposes. Voxels are displayed after cubic convolution resampling.

bioRxiv preprint doi: https://doi.org/10.64898/2026.09.25.754497; this version posted October 1, 2026. The copyright holder for this preprint (which was not certified by peer review) is the author/funder. This article is a US Government work. It is not subject to copyright under 17 USC 105 and is also made available for use under a CC0 license.

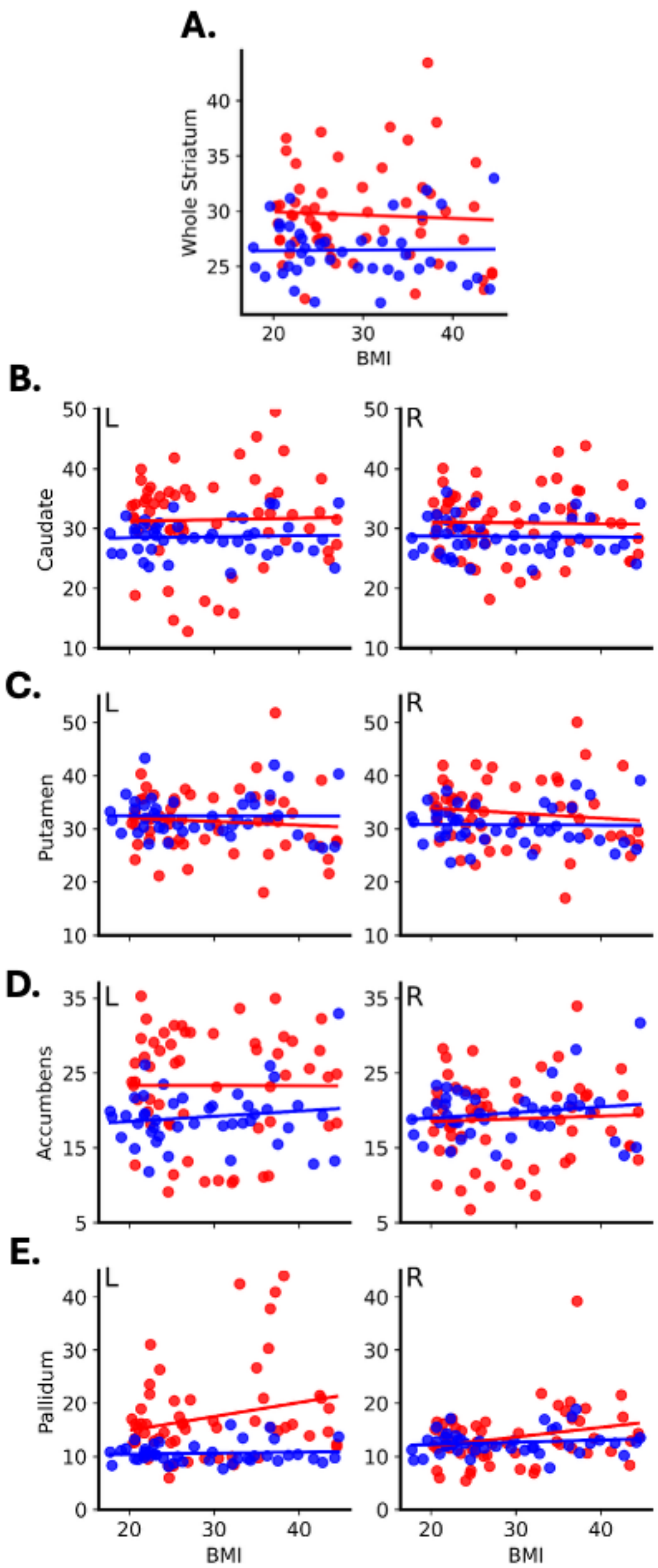


**Supplementary figure 5A-E. Similar relationship between BMI and D2BP in ROIs between fed and fasting states**. Fasted cohort (red); fed cohort (blue). Region of interest analysis comparing slopes of the relationship between D2BP as measured by [18F]fallypride and adiposity as measured by body mass index (BMI; kg/m2). Regression coefficients at the level of (A) the whole striatum were $\beta_{fed}$ vs. $\beta_{fasting}$ were 0.006 ± 0.074 vs. -0.029 ± 0.066 (p = 0.72). Regression coefficients across striatal subregions $\beta_{fed}$ vs. $\beta_{fasting}$ were as follows: (B) left caudate: 0.018 ± 0.121vs 0.025 ± 0.108 (p = 0.97); right caudate: -0.006 ± 0.094 vs. -0.015 ± 0.083 (p = 0.94); (C) left putamen: -0.002 ± 0.1 vs. -0.068 ± 0.089 (p = 0.62); right putamen: -0.008 ± 0.102 vs. -0.095 ± 0.091 (p = 0.52); (D) left accumbens: 0.07 ± 0.121 vs. -0.002 ± 0.107 (p = 0.66); right accumbens: 0.074 ± 0.095 vs. 0.036 ± 0.085 (p = 0.77); (E) left pallidum: 0.019 ± 0.127 vs. 0.263 ± 0.113 (p = 0.15); right pallidum: 0.042 ± 0.082 vs. 0.181 ± 0.073 (p = 0.21).

bioRxiv preprint doi: https://doi.org/10.64898/2026.09.25.754497; this version posted October 1, 2026. The copyright holder for this preprint (which was not certified by peer review) is the author/funder. This article is a US Government work. It is not subject to copyright under 17 USC 105 and is also made available for use under a CC0 license.

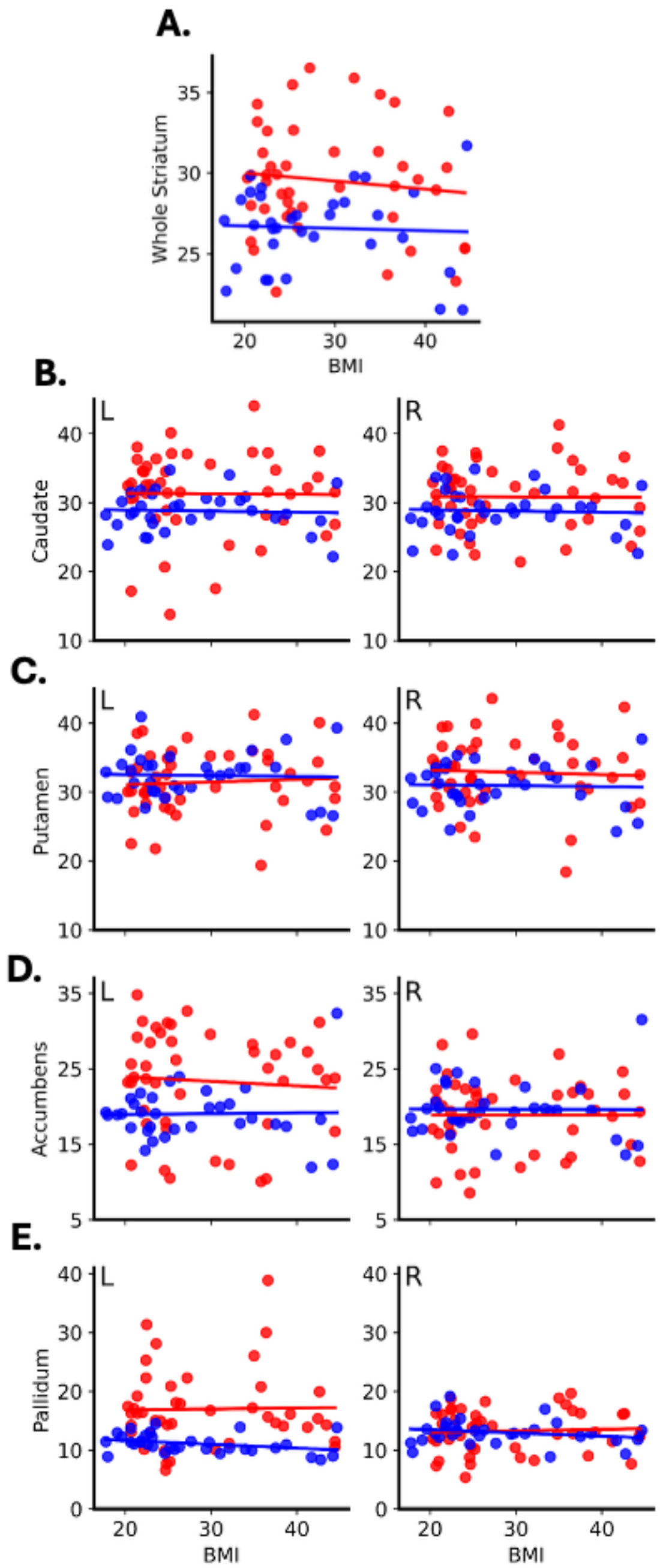


**Supplementary figure 6A-E. Similar relationship between BMI and D2BP in ROIs between cohorts in fed and fasting states, adjusted for age, sex and insulin sensitivity**. Fasted cohort (red); fed cohort (blue). Region of interest analysis comparing slopes of the relationship between D2BP as measured by [18F]fallypride and adiposity as measured by body mass index (kg/m2), adjusted for age, sex and insulin sensitivity (HOMA-IR). Regression coefficients at the level of (A) the whole striatum were $\beta_{fed}$ vs. $\beta_{fasting}$ were -0.001 ± 0.073 vs -0.029 ± 0.077 (p = 0.77). Regression coefficients $\beta_{fed}$ vs. $\beta_{fasting}$ across striatal subregions were as follows: (A) left caudate: 0.005 ± 0.121 vs. 0.029 ± 0.128 (p = 0.88); right caudate: -0.001 ± 0.099 vs. 0.027 ± 0.104 (p = 0.83); (B) left putamen -0.015 ± 0.102 vs. 0.04 ± 0.108 (p = 0.68); right putamen: -0.004 ± 0.106 vs. -0.019 ± 0.112 (p = 0.92); (C) left accumbens: 0.039 ± 0.136 vs. -0.013 ± 0.143 (p = 0.77); right accumbens: -0.011 ± 0.105 vs. -0.015 ± 0.11 (p = 0.98); (D) left pallidum: -0.008 ± 0.12 vs. 0.123 ± 0.126 (p = 0.41); and right pallidum: -0.052 ± 0.074 vs. 0.034 ± 0.078 (p = 0.38).

bioRxiv preprint doi: https://doi.org/10.64898/2026.09.25.754497; this version posted October 1, 2026. The copyright holder for this preprint (which was not certified by peer review) is the author/funder. This article is a US Government work. It is not subject to copyright under 17 USC 105 and is also made available for use under a CC0 license.

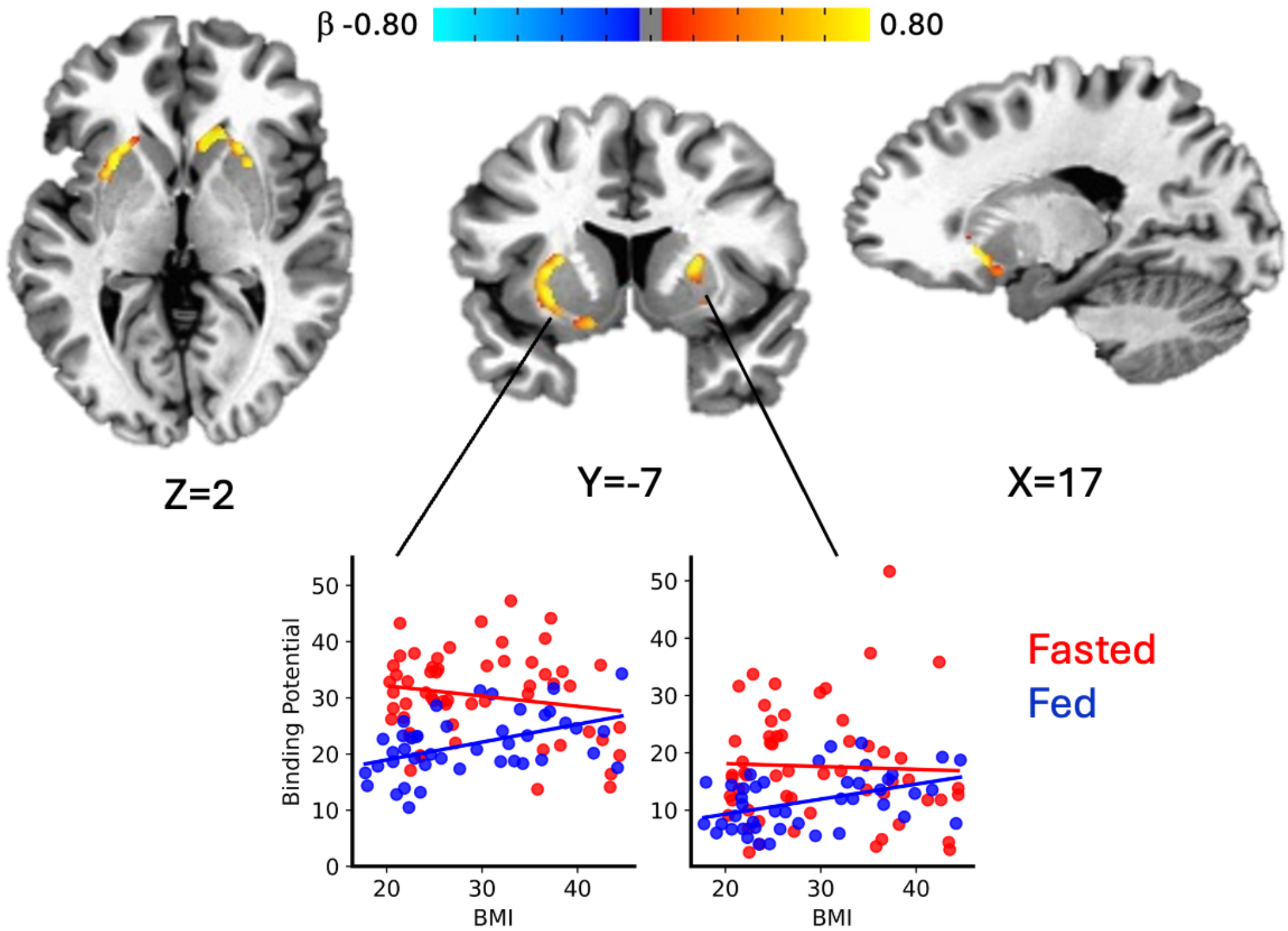


**Supplementary Figure 7. Voxelwise comparison of correlation between dopamine D2 binding potential and adiposity in Fed and Fasted cohorts, adjusted for age, sex, and insulin sensitivity.** Voxel-wise analyses within striatal mask identified clusters where the regression β coefficients of the D2BP-BMI relationship are significantly different in fed vs fasted cohorts in the left dorsolateral putamen (left plot) and in the right pallidum (right plot). Clusters are defined by faces-touching neighbor clustering, bi-sided uncorrected $p < 0.1$, cluster size > 30 voxels, and multiple-comparison corrected $p < 0.05$. Voxels are displayed after cubic convolution resampling. The color maps represent the difference of the regression β-coefficients between the fed and fasting states, displayed in cluster-specific scatter plots for visualization purposes.
